# Plastisphere-isolated *Stutzerimonas balearica* SP-H sustains stable sulfur-autotrophic denitrification under microplastic stress

**DOI:** 10.3389/fmicb.2026.1856465

**Published:** 2026-06-19

**Authors:** Shuai Liu, Zhuolin Yu, Jingli Pang, Xiuya Xu, Yezi Fang, Mengyao Xing, Jing Yan, Da Ouyang, Yating Luo, Haibo Zhang

**Affiliations:** 1 Sino-Spain Joint Laboratory for Agricultural Environment Emerging Contaminants of Zhejiang, College of Environmental and Resource Sciences, Zhejiang Agriculture and Forestry University, Hangzhou, China; 2 Zhejiang Key Laboratory of Soil Remediation and Quality Improvement, Zhejiang Agriculture and Forestry University, Hangzhou, China; 3 Ecological and Environmental Science and Research Institute of Zhejiang, Hangzhou, China

**Keywords:** c-di-GMP, EPS, microplastic tolerance, plastisphere, sulfur-autotrophic denitrification

## Abstract

Microplastic (MP) accumulation in constructed wetlands potentially threatens sulfur-autotrophic denitrification under low C/N conditions, yet the resilience of plastisphere-derived denitrifiers remains poorly understood. Here, we isolated and compared two denitrifying strains, a plastisphere isolate, *Stutzerimonas balearica* SP-H, and a conventional wetland isolate, *Castellaniella denitrificans* S0-H, under polyamide (PA) and polyethylene (PE) MP exposure. MPs severely impaired strain S0-H, causing nitrate removal inhibition (50.40–74.42%), nitrite accumulation (93.54–95.79%), elevated N_2_O production (57.24–94.32%), and reduced sulfate generation (38.94–66.95%). In contrast, strain SP-H maintained efficient nitrate depletion and stable sulfur oxidation under MP stress. Mechanistically, strain SP-H's superior tolerance involved a coordinated multi-layered defense: higher activities of Nar, Nir, and Sox, upregulated c-di-GMP, enhanced polysaccharide-rich extracellular polymeric substances (EPS) production, and preserved ATP levels. Collectively, habitat origin may determine MP resistance, and plastisphere-isolated strains like SP-H represent promising bioaugmentation agents to stabilize sulfur-based autotrophic denitrification in MP-impacted wastewater systems.

## Graphical Abstract

**Figure:**
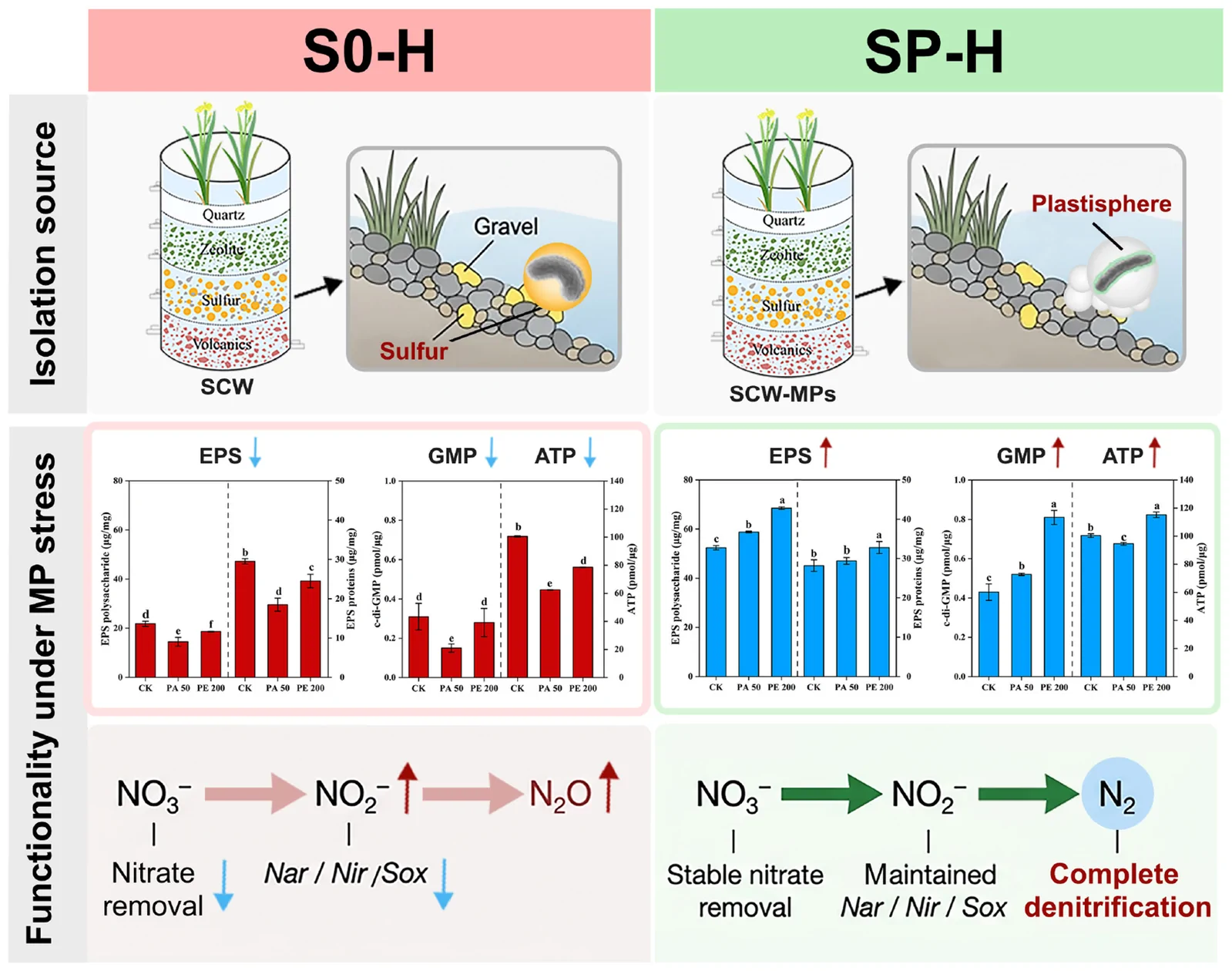

## 1 Introduction

Sulfur-autotrophic denitrification (SAD) is a critical pathway for nitrate removal in both natural and engineered aquatic environments, particularly under low carbon/ nitrogen (C/N) conditions (Shao et al., 2010). In this process, reduced sulfur compounds (e.g., thiosulfate, elemental sulfur) serve as electron donors driving reduction of nitrate (${\text{NO}}_{3}^{-}$) (Yuan et al., 2024). Unlike heterotrophic denitrification, SAD requires no external organic carbon and performs stable performance under carbon-limited conditions, making it attractive for wastewater treatment (Ma et al., 2020; Shao et al., 2024). The efficiency and stability of SAD depend on the coordinated activity of sulfur oxidation pathways (particularly the Sox system) and nitrogen reduction enzymes, including nitrate reductase (Nar) and nitrite reductase (Nir) (Feng et al., 2024; Chunyi et al., 2024). Disruption of these microbial-mediated electron transfer processes can lead to the accumulation of toxic intermediates such as nitrite (${\text{NO}}_{2}^{-}$) or the greenhouse gas nitrous oxide (N_2_O) (Su et al., 2022). Despite the common association of sulfur-oxidizers (e.g., *Thiobacillus* and *Sulfurimonas*) with SAD, other denitrifiers may contribute under specific conditions; their physiological roles, however, remain poorly characterized (Li et al., 2022; Wang et al., 2022b).

The rapid accumulation of microplastics (MPs) in aquatic ecosystems has emerged as a significant stressor that may impair these microbial functions (Kye et al., 2023; Jolaosho et al., 2025). MPs can interfere with denitrification through multiple pathways, including the generation of reactive oxygen species (ROS) leading to oxidative damage and physical disruption of cell membrane integrity (Sheridan et al., 2022; Wu et al., 2025) Recent community-level studies have shown that MP exposure can alter the abundance of nitrogen-transformation functional genes (Seeley et al., 2020; Li et al., 2024; Lin et al., 2024). Despite these findings, the specific physiological mechanisms by which MPs disrupt sulfur-nitrogen metabolic coupling in individual functional bacteria remain poorly understood.

In addition to acting as inhibitors, MPs can serve as artificial substrates for microbial colonization, forming a distinct ecological niche known as the “plastisphere” (Li et al., 2024; Malla et al., 2025; Wang and Sun, 2024). The plastisphere exposes microorganisms to long-term selective pressures that may drive ecological differentiation and favor specialized stress-response strategies (Battulga et al., 2024; Jia et al., 2024; Marques et al., 2023). These adaptive responses often involve remodeling of extracellular polymeric substances (EPS), enhanced biofilm formation, and regulation of c-di-GMP signaling, a key second messenger regulating surface colonization and stress resistance (Liu et al., 2024; Hindieh et al., 2025; Sun et al., 2024). Therefore, comparing the resilience of sulfur-autotrophic denitrifiers from different ecological niches, specifically the plastisphere vs. conventional microplastic-free environments, is essential for understanding microbial adaptation to emerging contaminants.

To address these knowledge gaps, two representative sulfur-based autotrophic denitrifying bacteria were isolated from distinct environmental niches: a conventional sulfur-based wetland system (strain S0-H) and a long-term MP-exposed plastisphere environment in a wetland system (strain SP-H). Using these two high-efficiency strains, we systematically investigated their physiological responses to polyamide (PA) and polyethylene (PE) MPs. Specifically, this study aimed to: (i) evaluate the effects of MP type and dosage on the growth and denitrification performance of sulfur-based autotrophic denitrifiers; (ii) compare the adaptive responses of strains originating from different ecological niches under MP stress; and (iii) elucidate the potential mechanisms underlying microbial adaptation, with particular emphasis on biofilm formation, EPS production, signaling regulation, and key enzyme activities involved in nitrogen and sulfur metabolism.

## 2 Materials and methods

### 2.1 Strain source, isolation, and screening

Microbial samples were collected from a laboratory-scale sulfur-based autotrophic constructed wetland simulation system that had been continuously operated for over 90 d. The system used a 1:1 (v/v) mixture of sulfur and limestone as the filter medium, and the influent was simulated nitrate-contaminated water. Samples were obtained from two representative habitats: a conventional wetland habitat (from the middle filler zone without MPs) and an MPs-attached biofilm habitat (collected from MPs at a concentration of 50 mg/L (Supplementary Figure 1 in the Supporting Information).

Strains were enriched using a modified ATCC 1,255 selective medium containing 5.0 ± 0.5 g/L Na_2_S_2_O_3_·5H_2_O as the sole electron donor and 2.0 ± 0.3 g/L NaNO_3_ as the sole electron acceptor. Enrichment was conducted in anaerobic serum bottles at 30°C and 150 rpm in the dark for 7 d, followed by purification using the standard streak plate method. From the isolated pure strains, two highly efficient strains, S0-H (from the conventional wetland) and SP-H (from the MP biofilm), were selected based on their superior nitrate removal efficiencies (>85% within 24 h) and stable sulfate production.

### 2.2 Morphological, physiological, and molecular identification of the isolated strains

Colony morphology was observed after 7 d of anaerobic cultivation at 30°C. Cellular morphology and size were examined using a Zeiss Sigma 300 field emission scanning electron microscope (SEM). Physiological and biochemical tests, including catalase and oxidase activities, salinity tolerance (0–8% w/v NaCl), optimal pH (5.0–9.0), and optimal temperature (25–45 °C), were conducted according to Bergey's Manual of Systematic Bacteriology (Supplementary Figure 2) (Weisburg et al., 1991). For molecular identification, genomic DNA was extracted using a bacterial DNA extraction kit (Tiangen Biochemical, DP302). The 16S rRNA gene was amplified with universal primers 27F and 1,492R (Weisburg et al., 1991), and the purified PCR products were sequenced. Phylogenetic analysis was performed by comparing the obtained sequences against the GenBank database using BLAST, and a phylogenetic tree was constructed using the Neighbor-Joining method with 1,000 bootstrap replicates in MEGA 7.0 software (Kumar et al., 2016). To assess the metabolic diversity of the two isolates, strains S0-H and SP-H were cultivated in a modified ATCC 1,255 medium (NaNO_3_ as the sole electron acceptor) supplemented with individual organic substrates (glucose, sucrose, sodium acetate, sodium citrate, methanol, and ethanol; 1.0 g/L) (Supplementary Table 1).

### 2.3 MPs stress experimental design

Two typical commercial MPs (purity >99.5%) were selected for the stress experiments: PA (125 ± 15 μm), PE (110 ± 20 μm). MPs were pretreated by immersing in 75% ethanol, rinsing with deionized water, and sterilizing by UV irradiation to remove possible organic contaminants and microbiological impurities. A modified ATCC 1,255 selective medium containing 2,500 mg/L Na_2_S_2_O_3_·5H_2_O as the sole electron donor and 50.0 mg/L NaNO_3_ as the sole electron acceptor. Experiments were conducted in 100 mL serum bottles containing 50 mL of medium under the predetermined optimal conditions for each strain. The experimental variables included strains (S0-H or SP-H), MP types (PA and PE) and concentrations (0, 50, and 200 mg/L). Each group was conducted in triplicate. Bottles were sealed with butyl rubber stoppers, purged with high-purity nitrogen gas for 5 min, and incubated at 30°C and 150 rpm in the dark. Samples were systematically collected at 0, 12, 24, 36, 48, 60, and 72 h for dynamic monitoring of multiple indicators. Strictly anaerobic conditions were verified by adding resazurin (1.0 mg/L) to each bottle as a redox indicator, the solution remained colorless throughout incubation, indicating no detectable oxygen ingress.

### 2.4 Analytical methods

For all liquid sample analyses, cultures were first centrifuged at 10,000 × g for 10 min at 4°C, and the supernatants were filtered through 0.22 μm polyethersulfone membrane filters (Jinjing, Tianjin, China) to remove residual bacterial cells and MP particles prior to quantification of chemical parameters. Nitrate (${\text{NO}}_{3}^{-}$) was determined using UV spectrophotometry (UV-2600i, Shimadzu, Kyoto, Japan), and nitrite (${\text{NO}}_{2}^{-}$) was detected using the N-(1-naphthyl)-ethylenediamine spectrophotometric method, following standard protocols. Nitrous oxide (N_2_O) was quantified using gas chromatography equipped with an electron capture detector (GC-ECD, Agilent 7,890B, Santa Clara, CA, USA). Sulfate (${\text{SO}}_{4}^{2-}$) concentrations were analyzed via Ion Chromatography (IC, ICS-6,000, Thermo Fisher Scientific, Waltham, MA, USA).

Dissolved oxygen (DO) was measured by inserting an optical DO probe (HQ40d/LDO10101, HACH, Loveland, CO, USA) through a rubber stopper into the liquid phase, pH and oxidation-reduction potential (ORP) were measured using respective electrochemical sensors (pH meter: FE28, Mettler Toledo, Columbus, OH, USA; ORP meter: SX712, Sanxin, Shanghai, China). Besides, dissolved organic carbon (DOC) derived from MPs (PA, PE MPs; 50, 200 mg/L) was quantified in sterile abiotic controls. Each group was conducted in triplicate. After incubation, the supernatant was filtered (0.22 μm membrane) and analyzed using a TOC analyzer (TOC-L CPH, Shimadzu, Kyoto, Japan).

Cell growth was monitored via OD_600_ using a UV-Vis spectrophotometer. Extracellular polymeric substances (EPS) were extracted using the formaldehyde-NaOH method. EPS polysaccharides were quantified by the anthrone-sulfuric acid method at 625 nm with glucose as the standard. EPS proteins were measured using a BCA protein assay kit (Solarbio, Beijing) at 562 nm with bovine serum albumin as the standard. Extracellular DNA (eDNA) was determined at 260 nm using a DNA assay kit based on the diphenylamine microplate method. Intracellular c-di-GMP concentrations were determined using LC-MS/MS (QTRAP 6500 +, SCIEX, Framingham, MA, USA).

Cell membrane integrity was visualized using confocal laser scanning microscopy (CLSM, Leica TCS SP8, Wetzlar, Germany) coupled with Live/Dead staining (BacLight^TM^ Bacterial Viability Kit, Thermo Fisher Scientific, Waltham, MA, USA). The activities of nitrate reductase (Nar), nitrite reductase (Nir), and sulfur oxidation enzyme complex (Sox), were quantitatively evaluated using established colorimetric methods with commercially available assay kits (Nar/Nir: Solarbio, Beijing, China; Sox: Meimian, Jiangsu, China) following the manufacturers' protocols. Cellular energy levels were assessed by measuring ATP content via a luciferase bioluminescence assay (ATP Assay Kit, Beyotime, Shanghai, China). Intracellular ROS levels were assessed using 2′,7′-dichlorofluorescein diacetate (DCFH-DA, Beyotime, Shanghai, China) as a fluorescent probe. After 48 h of incubation, cells were pelleted (8,000 rpm, 10 min, 4 °C), washed, and then incubated with 10 μM DCFH-DA (37 °C, 30 min). Following three washes with PBS, fluorescence was measured at 488/525 nm.

### 2.5 Statistical analysis

Data were presented as the mean value (± SD, *n* = 3) of three independent replicates. Statistical analysis was performed using two-way analysis of variance (ANOVA) with SPSS software (Version 22.0, IBM, Armonk, NY, USA), with a significance threshold of *p* < 0.05. Graphs were generated using Origin 2022 (OriginLab, Northampton, MA, USA).

## 3 Results

### 3.1 Morphological characteristics and denitrification performance of isolated strains

**Figure 1:**
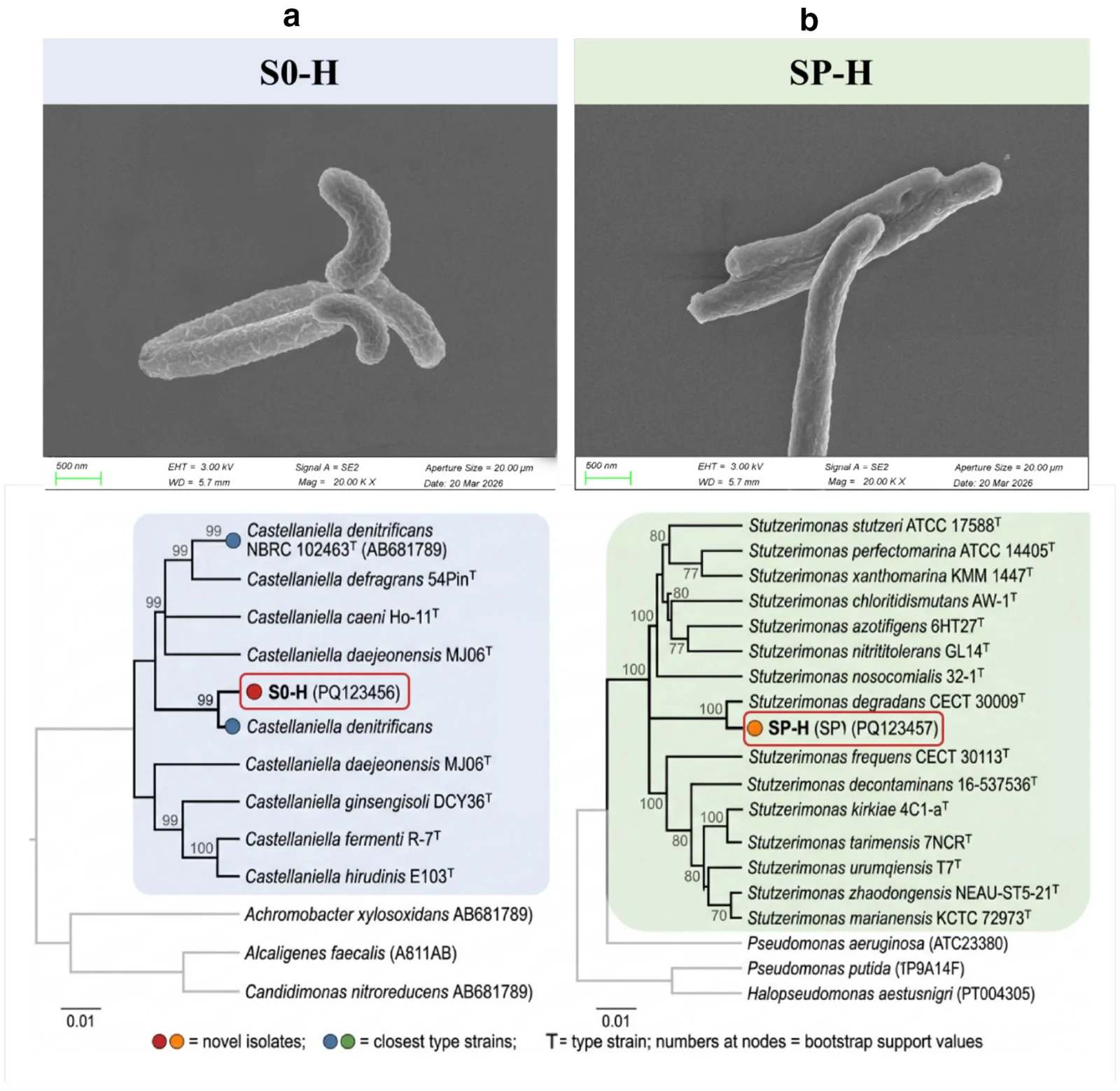
Morphology and phylogenetic identification of *Castellaniella denitrificans* S0-H **(a)** and *Stutzerimonas balearica* SP-H **(b)**. Bootstrap values (>50%) are shown at nodes, scale bar indicates substitutions per site.

Based on 16S rRNA gene sequencing and phylogenetic analysis, strain S0-H was identified as *Castellaniella denitrificans* (sequence similarity >99%), and strain SP-H was identified as *Stutzerimonas balearica* (sequence similarity >99%). SEM imaging revealed distinct cellular morphologies: S0-H exhibited a curved, elongated rod (vibrioid) morphology enmeshed within a loose, filamentous extracellular network (Figure 1a), whereas SP-H displayed a stout rod-shaped morphology with tight, dense cellular aggregation (Figure 1b). Both strains were catalase-positive and oxidase-negative, with optimal growth at pH 7.0–7.5 and 30–35°C, tolerating up to 3% (w/v) NaCl. Carbon source utilization profiling revealed distinct metabolic versatility between the two isolates. S0-H mainly utilized organic acids (e.g., sodium acetate, sodium citrate), whereas SP-H grew on a broader range of substrates (including sugars, alcohols, and organic acids) (Supplementary Table 1), suggesting potential for facultative mixotrophy in SP-H.

**Figure 2:**
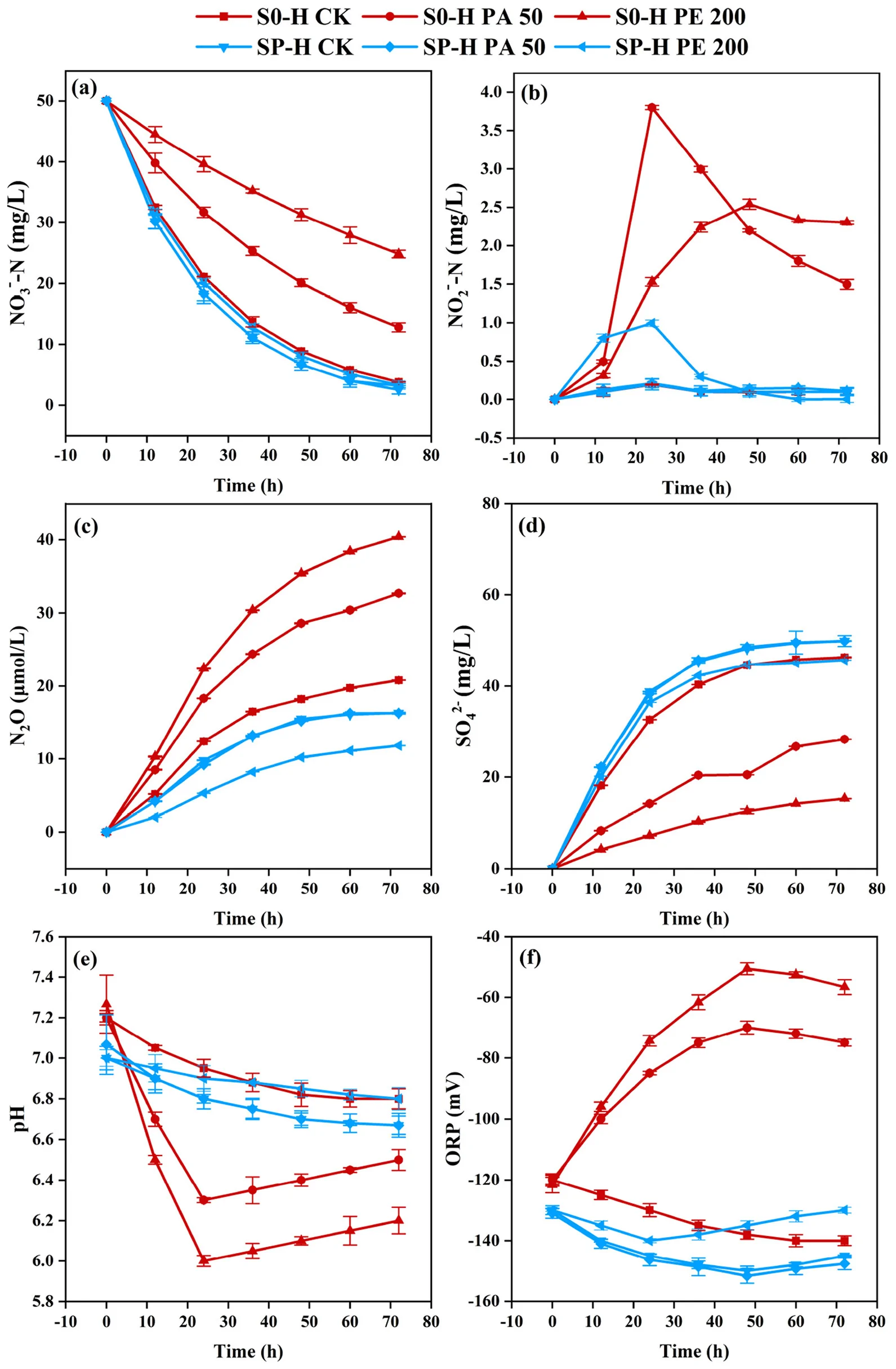
${\text{NO}}_{3}^{-}$-N **(a)**, ${\text{NO}}_{2}^{-}$-N **(b)**, N_2_O emission **(c)**, ${\text{SO}}_{4}^{2-}$ **(d)**, pH **(e)**, and ORP **(f)** in the strains S0-H and SP-H incubation system under MP stress. The error bars represent standard deviations (SD) (*n* = 3).

The denitrification performance of strains S0-H and SP-H exhibited distinct responses to MP exposure (PA and PE). In the control group (CK), both strains S0-H and SP-H exhibited rapid depletion of NO_3_^−^–N, with rates of 92.0 ± 2.0% and 94.0 ± 2.0%, respectively. However, the addition of MPs significantly inhibited the nitrate removal in strain S0-H (Figure 2a). At 72 h, the residual NO_3_^−^–N concentration in S0-H remained substantially higher in the PA 50 (12.84 ± 1.21 mg/L) and PE 200 (24.76 ± 1.95 mg/L) treatments than CK (3.82 ± 0.15 mg/L). In contrast, SP-H maintained efficient NO_3_^−^ depletion under both MP treatments (NO_3_^−^–N: CK, 2.49 ± 0.66 mg/L; PA 50, 3.16 ± 0.32 mg/L; PE 200, 3.29 ± 0.74 mg/L), and there were no significant differences among the three treated groups. Consistently, pronounced ${\text{NO}}_{2}^{-}$ accumulation was observed in S0-H under MP stress, particularly in the PA 50 treatment (reached 3.82 ± 0.85 mg/L at 24 h) (Figure 2b). In the PE 200 treatment, the accumulation of ${\text{NO}}_{2}^{-}$ persisted until the late stage of incubation (reached 2.3 ± 0.68 mg/L at 72 h). In contrast, ${\text{NO}}_{2}^{-}$ accumulation in SP-H was slight under both treatments. This indicated that strain SP-H possessed markedly higher functional stability to MP stress than strain S0-H.

A similar strain-dependent pattern was also observed for gaseous and sulfur-conversion products. MP exposure exacerbated N_2_O accumulation in the S0-H system, with the highest N_2_O content detected in the PE 200 treated group (reached 40.42 ± 1.25 μmol/L at 72 h), followed by the PA 50 treated group (reached 32.69 ± 0.85 μmol/L at 72 h) (Figure 2c). In contrast, SP-H maintained relatively low N_2_O production. Compared with the CK, PA 50 did not induce a significant increase and PE 200 even led to a marked decrease (reduced by 26.46 ± 0.92%) in N_2_O production. Meanwhile, ${\text{SO}}_{4}^{2-}$ production in S0-H was strongly suppressed by MPs. At 72 h, the ${\text{SO}}_{4}^{2-}$ concentrations in the PA 50 and PE 200 treatments decreased by 39.2 ± 1.23% and 67.2 ± 1.23%, respectively, compared to the CK group (Figure 2d). In contrast, the SP-H system exhibited stable and robust ${\text{SO}}_{4}^{2-}$ production, with concentrations reaching 49.87 ± 1.17 mg/L (no significant difference from CK: 49.90 ± 0.05 mg/L) and 45.70 ± 0.06 mg/L (only 8.4% reduction) under both PA 50 and PE 200 treatments at 72 h, consistent with its high ${\text{NO}}_{3}^{-}$removal efficiency. These results demonstrated that sulfur oxidation and denitrification performance were markedly suppressed in S0-H under MP stress, yet remained unaffected in SP-H.

Under MP stress, the ORP in the S0-H system shifted significantly to less reducing conditions (CK: −140.0 ± 2.4 mV; PA 50: −75.0 ± 1.7 mV: PE 200: −56.0 ± 1.3 mV at 72 h), whereas that of strain SP-H maintained a relatively stable and more strongly reductive environment (Figure 2f). Similarly, the pH in the S0-H system decreased significantly from 6.8 ± 0.2 to 6.5 ± 0.1 and 6.2 ± 0.3, whereas the pH of strain SP-H remained relatively stable throughout the incubation period (Figure 2e). Over 72 h, DO was consistently < 0.20 mg/L across all treatments in both systems, with no significant differences (*p* > 0.05; Supplementary Figure 3). These results suggested that MP stress exerted a stronger disruptive effect on nitrogen removal, sulfur oxidation, and microenvironmental stability (e.g., pH and ORP) in S0-H than in SP-H, indicating greater functional resistance of SP-H to MP stress.

### 3.2 Growth and key enzyme activities of strains S0-H and SP-H

**Figure 3:**
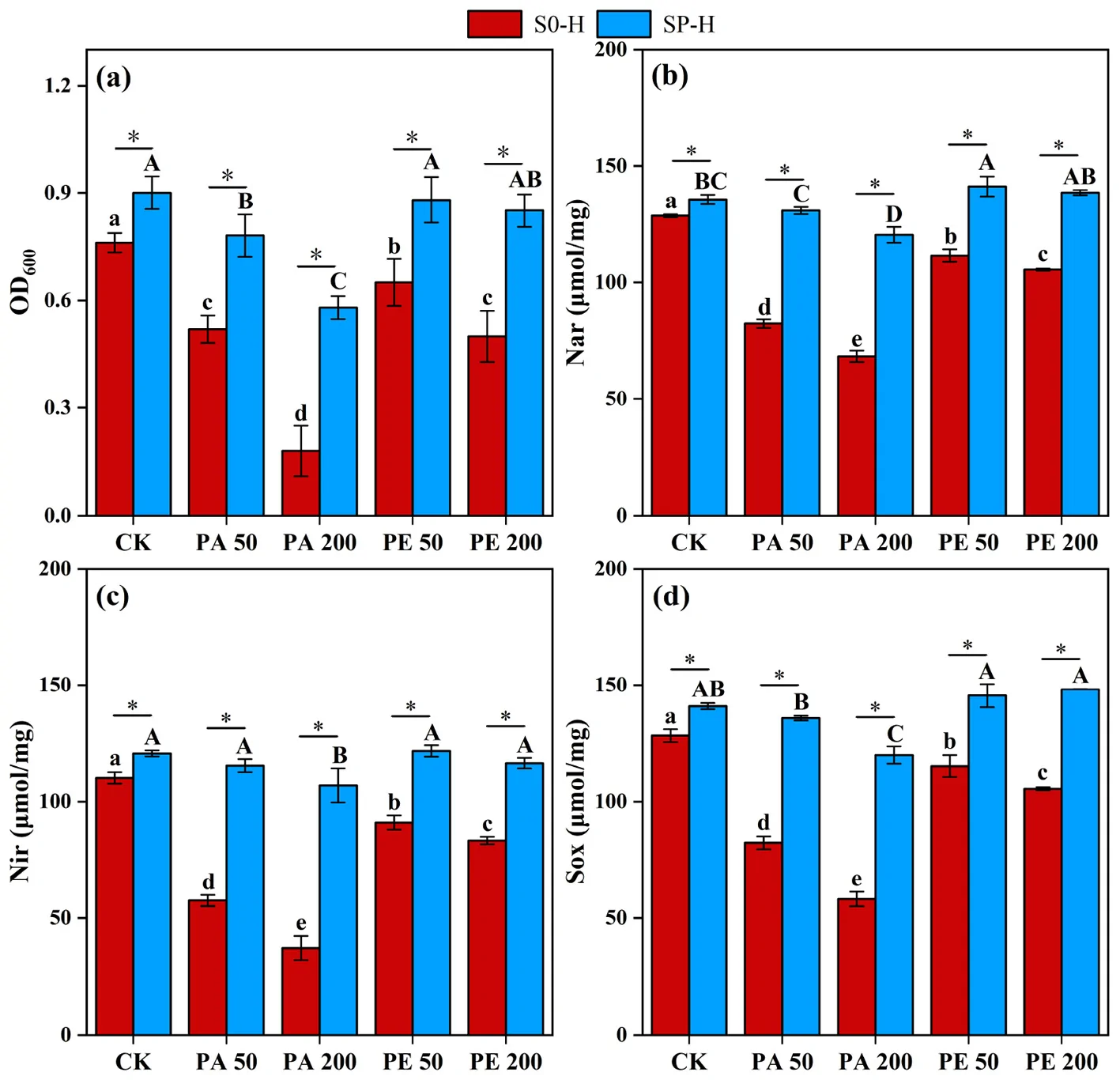
Effects of MPs on OD_600_ **(a)**, Nar **(b)**, Nir **(c)**, and Sox **(d)** activities of strains S0-H and SP-H. The error bars represent standard deviations (SD) (*n* = 3). Asterisks indicate significant differences among treatments (*, *p* < 0.05).

Compared with the CK, OD_600_ decreased in all PA treatments, with stronger inhibition at 200 mg/L than at 50 mg/L (Figure 3a). PA exerted a more pronounced inhibitory effect than PE at the same concentration, particularly for S0-H, whose OD_600_ dropped sharply from 0.76 ± 0.3 to 0.18 ± 0.05 under PA 200. In contrast, SP-H maintained higher OD_600_ values across all treatments, and PE did not significantly affect its growth (Figure 3a). These results demonstrated that the inhibitory effect of PA on microbial growth was stronger than that of PE, while SP-H exhibited greater MP tolerance than S0-H.

Under optimal conditions, both strains exhibited robust nitrate reductase (Nar; S0-H: 128.5 ± 2.32 μmol/mg; SP-H:136.9 ± 2.65 μmol/mg) and nitrite reductase (Nir; S0-H: 110.15 ± 1.86 μmol/mg; SP-H:120.64 ± 2.13 μmol/mg) activities (Figures 3b, c). Strain SP-H maintained significantly higher activities than S0-H under both control and MP-exposed conditions, indicating a stronger capacity to sustain nitrate and nitrite reduction under MP stress. This was consistent with the previous denitrification rate results of the two strains. In S0-H, both enzymes were markedly inhibited by MP concentration, with the strongest reduction observed under PA 200 (Nar: decreased by 46.6 ± 2.43%; Nir: decreased by 66.1 ± 1.96%) and PE 200 (Nar: decreased by 17.4 ± 0.84%; Nir: decreased by 24.7 ± 1.42%). In contrast, SP-H showed limited inhibition in Nar and Nir activities after MP exposure. Although both activities decreased under PA 200 (Nar: 120.4 ± 3.12 μmol/mg; Nir: 106.42 ± 1.25 μmol/mg) relative to the CK, they remained higher than those of S0-H (Nar: 51.74 ± 1.46 μmol/mg; Nir: 69.28 ± 1.32 μmol/mg) under the same condition.

A similar but more pronounced pattern was observed for sulfur oxidation enzyme (Sox) activity (Figure 3d). In S0-H, Sox activity decreased under both MP treatments, with the most pronounced reduction under PA 200 (70.86 ± 2.13 μmol/mg). By contrast, SP-H maintained consistently high Sox activity under MP stress (119.4 ± 2.52 μmol/mg). This suggested that the impact of MPs on sulfur oxidation-related enzyme activity was strain-dependent, which was more severely disrupted in S0-H than in SP-H under MP stress, further supporting the greater resilience of SP-H.

### 3.3 EPS composition, eDNA release, and c-di-GMP signaling

**Figure 4:**
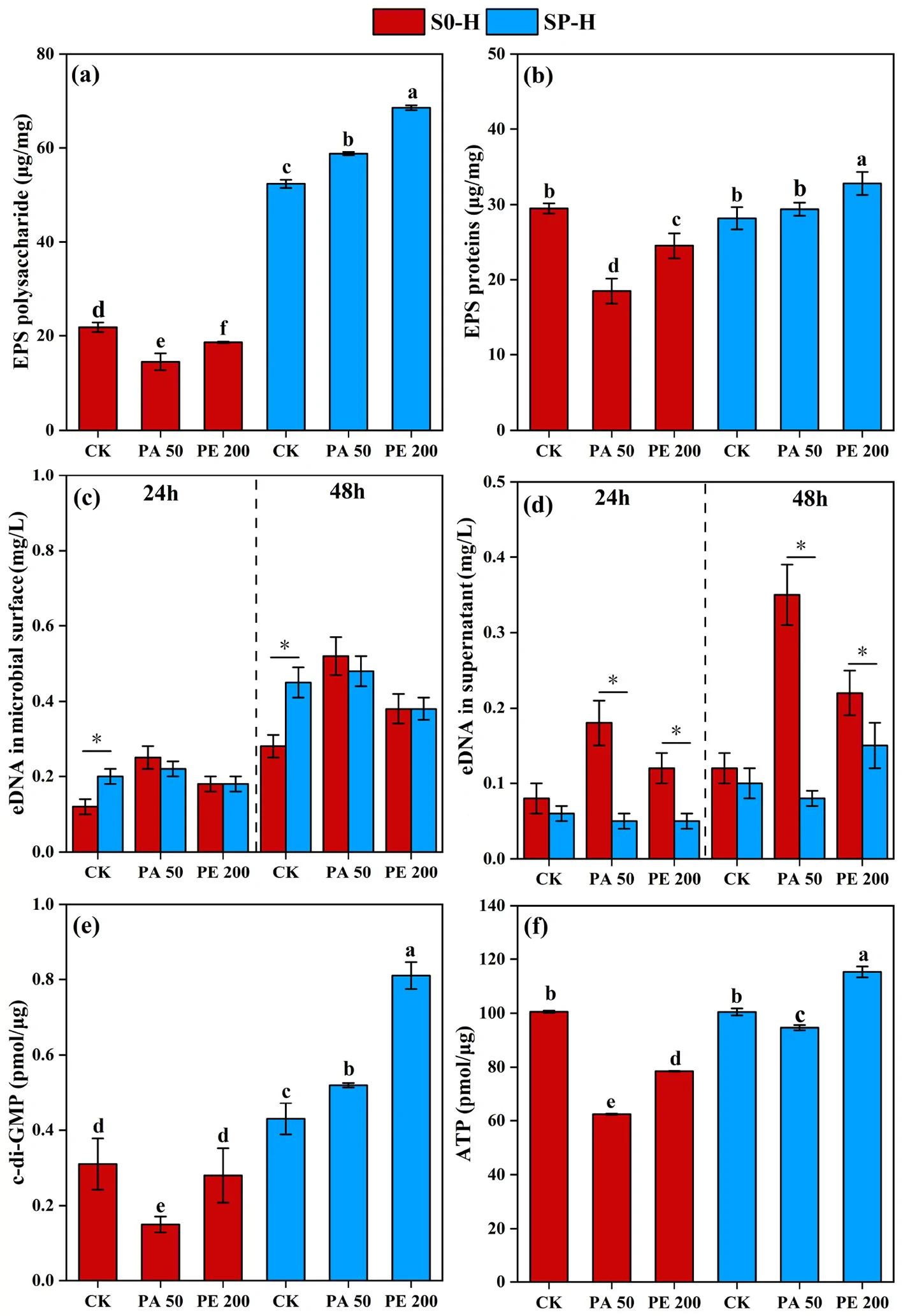
Effects of MPs on EPS polysaccharide **(a)**/proteins **(b)**, eDNA in microbial surface **(c)**, and supernatant **(d)**, c-di-GMP **(e)**, and ATP **(f)** concentration of strains S0-H and SP-H. The error bars represent standard errors of the mean (SD) (*n* = 3). Asterisks indicate significant differences among treatments (*, *p* < 0.05). Different lowercase letters indicate significant differences between different factors. Lowercase letters indicate significant differences among treatments within strain S0-H, and uppercase letters indicate significant differences among treatments within strain SP-H (*p* < 0.05).

EPS play a critical role in bacterial surface attachment and stress tolerance. Under MP stress, SP-H significantly upregulated EPS production in a dose-dependent pattern, with a pronounced increase (PE: 1.31 ± 0.86-fold; PA: 1.12 ± 0.43-fold) in polysaccharide-rich matrix components (*p* < 0.05) (Figures 4a, b). This robust EPS secretion produced a dense hydration shell around the cells, which may sterically block direct contact with sharp MP particles. In contrast, EPS production in S0-H was severely suppressed under MP exposure, with major EPS components declining significantly under both PA (decreased by 37.3 ± 0.53% and 33.5 ± 0.44%) and PE stress (decreased by 14.7 ± 0.32% and 17.1 ± 0.28%).

Consistent with these observations, the concentration of extracellular DNA (eDNA), an indicator of cellular lysis or EPS instability, was substantially higher in S0-H culture supernatant under MP stress, particularly in the PA 50 treatment (PA 50: 0.52 ± 0.05 mg/L; CK: 0.28 ± 0.03 mg/L) (Figures 4c, d). In contrast, eDNA levels in SP-H culture supernatant remained comparatively stable across treatments (0.48 ± 0.04 mg/L). These results suggested that S0-H possessed a structurally fragile EPS matrix prone to disintegration under MP stress, whereas SP-H maintained a stable, protective EPS barrier.

C-di-GMP is a key regulator of biofilm formation and EPS production. Under unstressed conditions, SP-H (0.43 ± 0.02) exhibited a higher baseline c-di-GMP level than S0-H (0.31 ± 0.03) (Figure 4e). Upon MP exposure, the two strains showed opposite regulatory responses: c-di-GMP was significantly downregulated in S0-H under PA stress (from 0.31 to 0.15 pmol/μg), whereas SP-H actively upregulated c-di-GMP expression, peaking at 0.81 pmol/μg under PE 200 stress, indicating a rapid transition toward a protective, surface-associated strategy. This likely contributed to the divergent EPS and eDNA profiles observed in the two strains.

### 3.4 ATP dynamics, ROS levels, and cell viability

The divergence in c-di-GMP signaling and EPS structure was tightly coupled with intracellular energy reserves and oxidative stress responses. Strain S0-H suffered severe ATP depletion under MP stress, with ATP content dropping from 100.52 ± 0.43 to 62.38 ± 0.19 pmol/μg under PA 50 exposure (Figure 4f), reflecting the disruption of its energy generation pathways. In contrast, strain SP-H maintained robust ATP levels under PA 50 stress and even significantly increased its ATP pool from 100.48 ± 0.13 to 115.25 ± 1.98 pmol/μg under PE 200 exposure, providing the thermodynamic foundation for its high-cost defensive responses.

**Figure 5:**
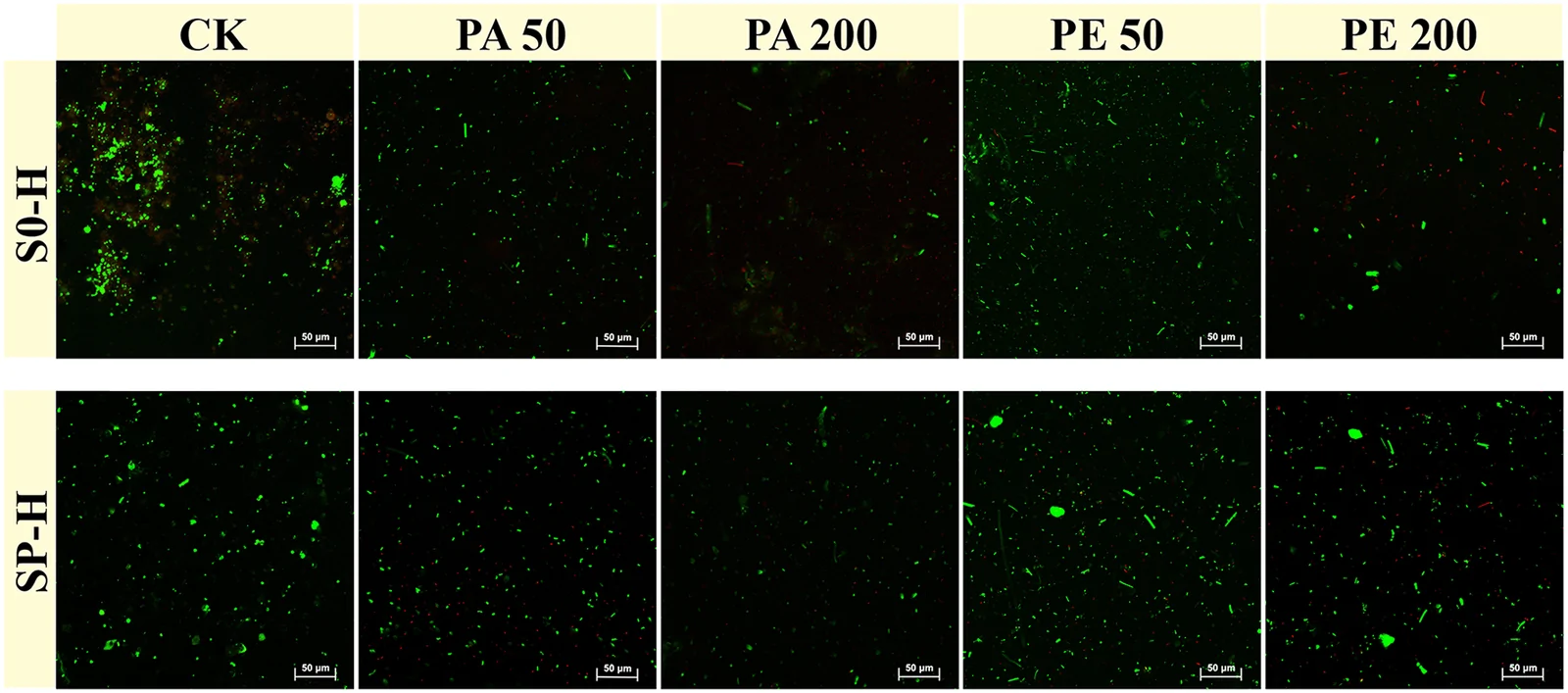
Confocal laser scanning microscopy (CLSM) images of strains S0-H and SP-H under PE and PA treatments. Green fluorescence: viable cells; red fluorescence: dead cells; yellow fluorescence: co-localization of viable and dead cells.

Meanwhile, ROS levels were elevated in MPs-treated groups, with S0-H (458.43 ± 35.67 RFU) exhibiting a higher oxidative stress status than SP-H (235.35 ± 22.14 RFU) under PA 200 treatments, indicating superior antioxidative defense in the plastisphere-isolated strain (SP-H) (Table 1). This oxidative imbalance likely contributed to membrane damage, as directly visualized by CLSM (Figure 5). Quantitative cell viability analysis confirmed this divergence: the viable cell proportion in S0-H decreased from 92.4 ± 2.1% (CK) to 38.6 ± 4.5% under PA 200, whereas SP-H maintained 78.3 ± 3.1% viability under the same stress (Supplementary Table 2), consistent with the ROS, ATP, and eDNA results. Under MP stress, SP-H maintained predominantly viable cells (green fluorescence), while S0-H exhibited a marked increase in red fluorescence (dead/damaged cells), particularly under PA exposure. Quantitative ROS analysis further confirmed that S0-H suffered more extensive membrane damage than SP-H, which likely contributed to intracellular component leakage and subsequent metabolic decline. Collectively, it was inferred that SP-H's superior MP tolerance was mediated by coordinated energy maintenance, antioxidative capacity, and membrane integrity.

**Table 1:** ROS levels of two strains under MP stress.

Treatment	S0-H ROS (RFU)	SP-H ROS (RFU)
CK	102.15 ± 8.34	105.54 ± 10.63
PA 50	325.46 ± 28.64^**^	158.76 ± 15.31^*^
PA 200	458.43 ± 35.67^**^	235.35 ± 22.14^**^
PE 50	118.34 ± 10.56	112.63 ± 11.67
PE 200	198.48 ± 18.41^*^	142.21 ± 12.75^*^

Values represent means with SD (*n* = 3). Asterisks indicate significant differences between each-MP treatment and·the corresponding control (CK) of the same strain (^*^, *p* < 0.05; ^**^, *p* < 0.01). Abbreviations: S0-H, *Castellaniella denitrificans*; SP-H, *Stutzerimonas balearica*.

## 4 Discussion

### 4.1 Strain-dependent denitrification stability under MP stress

In this study, we examined sulfur-autotrophic denitrifying bacteria from two habitats: a microplastic-free wetland (S0-H) and a microplastic-colonized biofilm (SP-H). Upon MP exposure, their functional and morphological differences were assessed, revealing habitat-dependent tolerance. Beyond the morphological distinction observed by SEM, the more ecologically meaningful difference between the two isolates was their strain-specific functional response to MP stress (Figure 1). MPs caused a pronounced functional deterioration in S0-H: residual ${\text{NO}}_{3}^{-}$-N at 72 h increased from 3.82 ± 0.15 mg/L in the CK to 12.84 ± 1.21 mg/L (PA 50) and 24.76 ± 1.95 mg/L (PE 200), whereas SP-H remained unaffected. S0-H also accumulated substantially more nitrite and N_2_O, with N_2_O reaching 40.42 ± 1.25 μmol/L under PE treatment at 72 h, while ${\text{SO}}_{4}^{2-}$ production was reduced by 39.2 ± 1.23% under PA 50 and 67.2 ± 1.23% under PE treatment (Figure 2b). The elevated N_2_O accumulation in S0-H implied that MPs stress may transform an efficient denitrifying strain into a system with greater greenhouse gas emission potential, particularly when sulfur oxidation and denitrification are functionally uncoupled (Su et al., 2022). Similar impairments of microbial activity and nitrogen transformation under chronic plastic exposure have also been reported in wastewater-associated systems (Wang et al., 2023).

The concomitant changes in ORP and pH further supported this interpretation. By 72 h, the S0-H culture shifted from a strongly reducing state (−140.0 ± 2.4 mV in the CK) to a much weaker reducing environment under MP exposure (PA 50: −75.0 ± 1.7 mV; PE 200: −56.0 ± 1.3 mV), accompanied by a decrease in pH from 6.8 ± 0.2 to 6.5 ± 0.1 and 6.2 ± 0.3 (Figures 2e, f). In contrast, SP-H maintained a comparatively stable redox regime and pH throughout incubation. As DO remained below 0.20 mg/L and showed no difference across treatments (Supplementary Figure 3), the ORP and pH shifts reflect strain-specific alterations in sulfur-nitrogen coupling, not differential oxygen intrusion. Such physicochemical stability was likely essential for sustaining sulfur oxidation and continuous electron transfer during autotrophic denitrification. Given that SP-H was isolated from a long-term MP-exposed biofilm, its superior functional stability likely reflected habitat-driven pre-adaptation. This was consistent with the ecological memory hypothesis and the view of the plastisphere as a stringent environmental filter, where polymer-specific properties (e.g., surface hydrophobicity, plastic-derived additives) exert selective pressures that drive microbial niche partitioning (Khalighi et al., 2022; Li et al., 2024; Sun et al., 2023). Through long-term exposure, strains such as SP-H have acquired stress-tolerance traits that enable them to flourish under conditions that suppress conventional isolates like S0-H, consistent with previous reports that long-time plastic exposure reshapes microbial activity and community structure (Wang et al., 2019, 2022a).

### 4.2 MP-induced growth inhibition and enzymatic disruptions in denitrification

SP-H exhibited higher tolerance to MPs than S0-H. The biomass of S0-H was significantly inhibited by PA200 (reduced by 76.32%) and PE200 (reduced by 34.21%), whereas under PA200, SP-H biomass decreased by only 35.56% (not significantly suppressed by PE200). This greater tolerance may be partly associated with its broader substrate utilization spectrum, which could enable partial co-utilization of PA/PE-derived organic carbon (Supplementary Figure 4). Across MP treatments, PA exerted a stronger inhibitory effect on biomass accumulation than PE, and this pattern was most evident in S0-H, whose OD_600_ decreased from 0.76 ± 0.30 (CK) to 0.18 ± 0.05 (PA 200) (Figure 3a). This polymer-specific inhibition was consistent with the view that MP surface polarity, hydrophobicity, and interfacial behavior govern bacterial-particle interactions (Sun et al., 2023; Huang et al., 2025). The higher polarity of PA likely caused stronger interference with the cell envelope and a structurally weaker EPS layer, whereas PE may have imposed less disruptive contact, particularly in strains better adapted to hydrophobic surfaces or capable of utilizing leached organic substrates (Corsaro et al., 2021; Zeng et al., 2023).

The enzyme data provided a mechanistic explanation for this strain-specific difference. Under control conditions, SP-H exhibited slightly higher Nar and Nir activities than S0-H (Nar: 136.9 ± 2.65 vs. 128.5 ± 2.32 μmol/mg; Nir: 120.64 ± 2.13 vs. 110.15 ± 1.86 μmol/mg), and it retained high activities even under PA 200 (Nar, 120.4 ± 3.12 μmol/mg; Nir, 106.42 ± 1.25 μmol/mg) (Figures 3b, c). By contrast, S0-H showed clear inhibition of Nar, Nir, and Sox after MP exposure, with Sox activity declining to 70.86 ± 2.13 μmol/mg under PA 200, whereas SP-H maintained a much higher Sox activity of 119.4 ± 2.52 μmol/mg under MP stress (Figure 3d). It was inferred that sulfur-based autotrophic denitrification depended on tight coupling between sulfur oxidation and stepwise nitrogen reduction, therefore, the inhibition of Sox was expected to constrain electron supply to Nar and Nir, thereby promoting nitrite accumulation and N_2_O release (Su et al., 2022).

### 4.3 EPS remodeling and c-di-GMP-mediated structural defense under MP exposure

Under MP exposure, SP-H increased EPS production in a dose-dependent manner and enriched its polysaccharide-rich matrix components by 1.12 ± 0.43-fold (PA) and 1.31 ± 0.86-fold (PE), whereas S0-H showed an overall decline in its major EPS fractions, with reductions of 37.3 ± 0.53% and 33.5 ± 0.44% under PA and 14.7 ± 0.32% and 17.1 ± 0.28% under PE (Figure 4a). This contrast suggested that SP-H actively reinforced its extracellular barrier under stress, while the EPS matrix of S0-H became destabilized. A polysaccharide-enriched matrix can enhance water retention, buffer local physicochemical fluctuations, and reduce direct particle-cell contact, thereby protecting cells from external damage (Moyal et al., 2023). Consistently, eDNA in S0-H increased from 0.28 ± 0.03 mg/L in the CK to 0.52 ± 0.05 mg/L under PA 50, whereas SP-H remained comparatively stable at approximately 0.48 ± 0.04 mg/L (Figures 4c, d).

The c-di-GMP responses provided a plausible regulatory basis for this divergence. C-di-GMP is a key signaling molecule that promotes cell attachment, EPS secretion, and biofilm formation (Kumar and Spiro, 2017). The higher basal c-di-GMP level in SP-H (SP-H: 0.43 ± 0.02 vs S0-H: 0.31 ± 0.03 pmol/μg), together with its further upregulation under MP exposure (0.81 pmol/μg under PE 200), suggested that this strain rapidly activated a surface-associated protective program characterized by stronger matrix production and stress buffering capacity (Chua et al., 2015; Valentini and Filloux, 2016). In S0-H, by contrast, c-di-GMP decreased markedly under PA stress, reduced from 0.31 pmol/μg to 0.15 pmol/μg, which suggested that the signaling network required to trigger matrix reinforcement was impaired and therefore unable to support an effective structural defense (Sun et al., 2022). Collectively, these results indicated that c-di-GMP-driven EPS remodeling was a key factor of strain-specific tolerance to MP exposure.

The eDNA data provided complementary evidence for EPS structural integrity differences between the two strains. In S0-H, eDNA increased by 86.0% under PA 50 stress (from 0.28 ± 0.03 to 0.52 ± 0.05 mg/L), consistent with accelerated cell lysis or EPS structural disintegration releasing nucleic acids into the extracellular environment (Turnbull et al., 2016). Although eDNA itself can serve as a structural scaffold in nascent biofilms under some circumstances (Nagasawa et al., 2020), the concomitant decline in EPS polysaccharide and protein fractions in S0-H indicated that eDNA accumulation reflected cellular damage rather than a deliberate secretory response. In contrast, SP-H maintained stable eDNA levels (approximately 0.48 ± 0.04 mg/L) across all treatments, consistent with preserved cell viability as confirmed by CLSM imaging (Figure 5). Collectively, these results demonstrated c-di-GMP-driven EPS remodeling as a key adaptive mechanism underlying strain-specific MP tolerance in sulfur-based autotrophic denitrifiers.

### 4.4 Energy homeostasis, oxidative stress, and membrane integrity

Intracellular energy status and oxidative stress levels are critical determinants of metabolic stability of strains under MP stress. In S0-H, ATP content decreased by −38% (from 100.52 ± 0.43 to 62.38 ± 0.19 pmol/μg) upon PA 50 exposure (Figure 4f). In contrast, SP-H maintained high ATP levels under PA 50 condition and even increased under PE 200 (from 100.48 ± 0.13 to 115.25 ± 1.98 pmol/μg). Given that Sox activity, the primary ATP-generating step in sulfur-based autotrophic metabolism, was simultaneously suppressed in S0-H, the ATP depletion in S0-H likely reflected an energetic cascade: impaired thiosulfate oxidation reduces proton motive force for ATP synthesis, limiting energy for membrane repair, EPS secretion, and antioxidant defense, thereby amplifying vulnerability to further MPs-induced damage (Imlay, 2013). Conversely, the ability of SP-H to sustain elevated ATP levels under MP stress indicated a sufficient metabolic foundation for sustained biochemical activity.

ROS data further confirmed that oxidative stress was a major driver of functional collapse in S0-H. Under PA 200 exposure, ROS level in S0-H reached 458.43 ± 35.67 RFU (−4.5-fold above control), whereas SP-H exhibited a moderate elevation (−2.2-fold above control) (Table 1). Under PE 200 treatment, ROS levels in S0-H and SP-H were 198.48 ± 18.41 and 142.21 ± 12.75 RFU, respectively. Excessive ROS is known to trigger lipid peroxidation, protein inactivation, and DNA damage, ultimately leading to metabolic collapse (Imlay, 2013). In S0-H, the concurrent ATP depletion and ROS elevation suggested a self-reinforcing deterioration cycle of energy deficiency-oxidative damage-membrane disruption (Figure 4f) (Cuthbertson et al., 2010).

In contrast, SP-H consistently maintained higher ATP levels and lower ROS accumulation under MP stress, indicating that this strain sustained cellular homeostasis through coordinated interplay between extracellular defenses and intracellular metabolic regulation (Sun et al., 2023; Huang et al., 2025). These findings collectively demonstrated that SP-H's superior MP tolerance relied on the synergistic integration of c-di-GMP signaling, EPS-mediated barrier reinforcement, sustained energy supply, and membrane protection (Imlay, 2013). For sulfur-autotrophic denitrifying bacteria, such multi-tiered cooperative adaptive capacity constitutes the fundamental basis for maintaining continuous N-removal performance under MP stress (Obana et al., 2020).

Our findings highlighted the potential of strain-targeted bioaugmentation in sulfur-based autotrophic constructed wetland simulation systems. Introducing MP-tolerant strains such as SP-H, possibly combined with suitable carriers, might enhance system robustness against MP stress (Seeley et al., 2020). Biodegradable polymers (e.g., polyhydroxyalkanoates) could serve as sustainable carrier alternatives, minimizing secondary MP risks while potentially providing auxiliary carbon sources (Li et al., 2022). Preliminary mesocosm experiments (Supplementary Figure 5) supported the strain-specific nature of this strategy: SP-H inoculation improved nitrogen removal and reduced N_2_O emissions under PA loading, whereas the conventional isolate S0-H did not confer comparable benefits. These results suggest a promising direction for future engineering applications, though further validation under varying operational conditions is needed.

## 5 Conclusion

The conventional wetland isolate *Castellaniella denitrificans* S0-H exhibited pronounced MP-induced impairments, including inhibited growth, suppressed activities of Nar, Nir, and Sox, uncoupling of sulfur oxidation and denitrification, substantial ${\text{NO}}_{2}^{-}$ accumulation, and elevated N_2_O emission. In contrast, the plastisphere-isolated strain *Stutzerimonas balearica* SP-H maintained high denitrification efficiency and sulfur oxidation stability under both PA and PE exposure, highlighting the plastisphere as an underexplored reservoir of stress-tolerant functional microorganisms. Mechanistically, SP-H's superior MP tolerance reflected a multi-layered cooperative strategy: (i) upregulation of c-di-GMP signaling, triggering (ii) enhanced production of polysaccharide-rich EPS that forms a protective extracellular barrier; (iii) sustained ATP levels and mitigated oxidative stress; and (iv) robust activities of key denitrifying and sulfur-oxidizing enzymes. Overall, plastisphere-isolated strain SP-H combines exceptional MP tolerance with complete denitrification, making it a promising bioaugmentation candidate for sulfur-based autotrophic denitrification systems under long-term MP exposure, especially in low C/N ratio wastewater treatment systems.

## Data Availability

The raw data supporting the conclusions of this article will be made available by the authors, without undue reservation.
