# Correction: Plastisphere-isolated *Stutzerimonas balearica* SP-H sustains stable sulfur-autotrophic denitrification under microplastic stress

**DOI:** 10.3389/fmicb.2026.1948177

**Published:** 2026-08-20

**Authors:** Shuai Liu, Zhuolin Yu, Jingli Pang, Xiuya Xu, Yezi Fang, Mengyao Xing, Jing Yan, Da Ouyang, Yating Luo, Haibo Zhang

**Affiliations:** 1 Sino-Spain Joint Laboratory for Agricultural Environment Emerging Contaminants of Zhejiang, College of Environmental and Resource Sciences, Zhejiang Agriculture and Forestry University, Hangzhou, China; 2 Zhejiang Key Laboratory of Soil Remediation and Quality Improvement, Zhejiang Agriculture and Forestry University, Hangzhou, China; 3 Ecological and Environmental Science and Research Institute of Zhejiang, Hangzhou, China

**Keywords:** c-di-GMP, EPS, microplastic tolerance, plastisphere, sulfur-autotrophic denitrification

In the original article, there was a mistake in Figure 5 as published. During the assembly of Figure 5, a preliminary layout containing provisional images was inadvertently uploaded instead of the final, correctly composed version. The corrected Figure 5 appears below.

**Figure 5:**
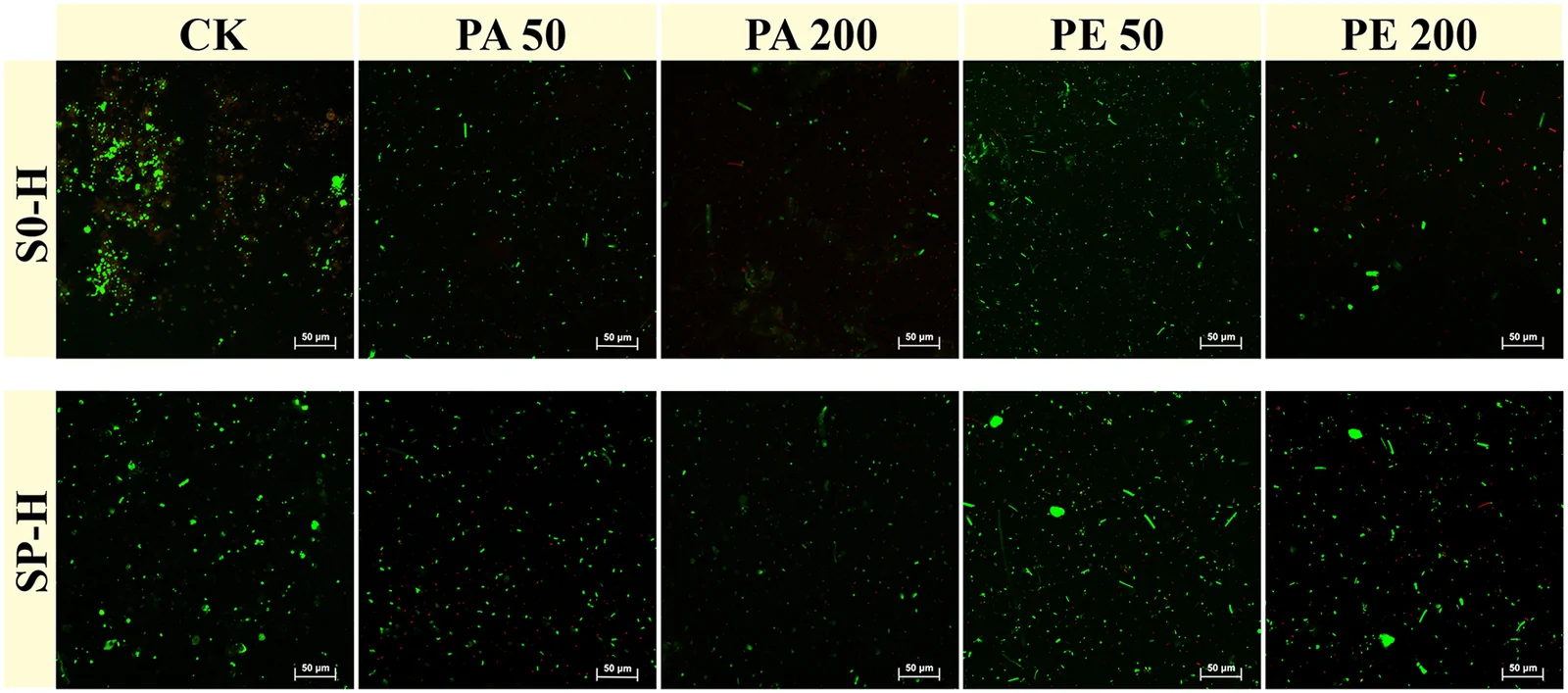
Confocal laser scanning microscopy (CLSM) images of strains S0-H and SP-H under PE and PA treatments. Green fluorescence: viable cells; red fluorescence: dead cells; yellow fluorescence: co-localization of viable and dead cells.

The original version of this article has been updated.

