# Supplementary material for: Plastisphere-isolated Stutzerimonas balearica SP-H sustains stable sulfur-autotrophic denitrification under microplastic stress
Source: Front Microbiol. 2026 Jun 19;17:1856465. doi: 10.3389/fmicb.2026.1856465 (PMC13328504; doi:10.3389/fmicb.2026.1856465)
Supplement: Supplementary file 1 [file Data_Sheet_1.docx]

**
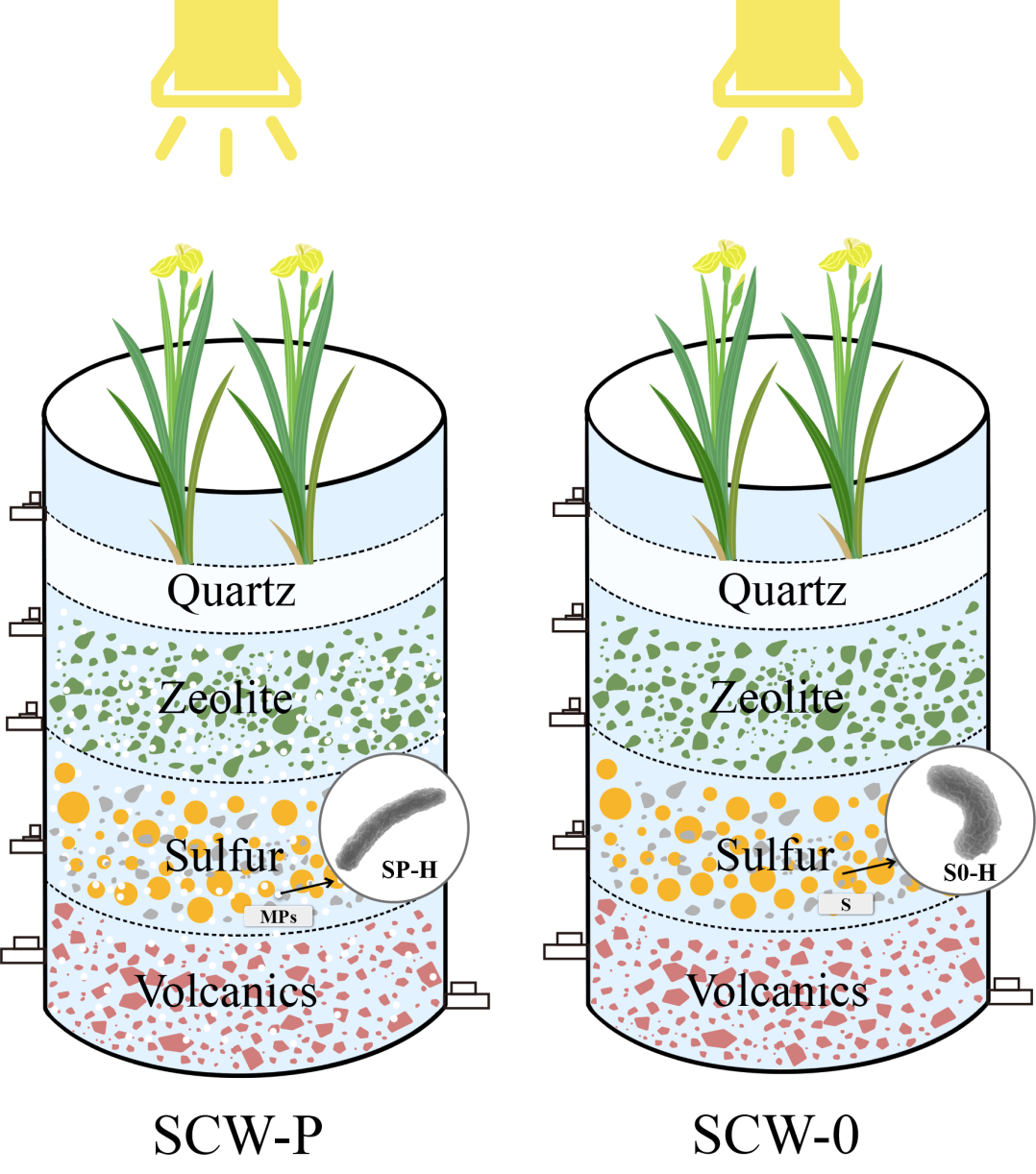
**

**Fig. S1** Schematic diagram of the sampling locations for strain isolation within the sulfur-based autotrophic constructed wetland simulation system. The strain S0-H was isolated from the middle sulfur filler zone without MP addition (SCW-0), while the strain SP-H was obtained from biofilms attached to PA/PE MPs (50 mg/L) in the same system (SCW-P).


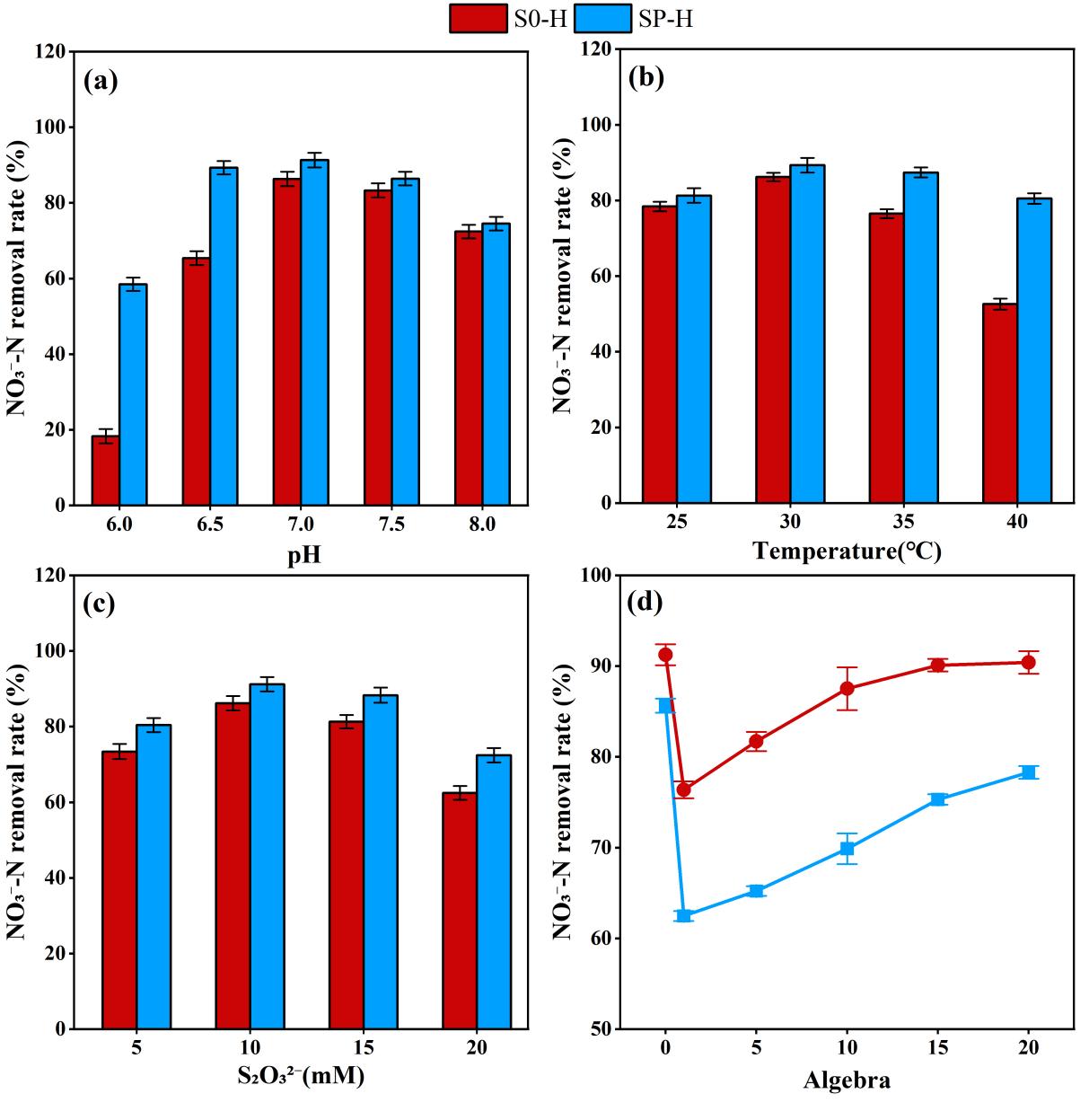


**Fig. S2** Denitrification activity responses of strains S0-H and SP-H to different pH (a), temperatures (b), and (c) S_2_O_3_^2-^, and their acclimation curves (d) under PA MP stress. The error bars represent standard deviations (SD) (*n*=3). Different letters indicate significant differences between different factors.


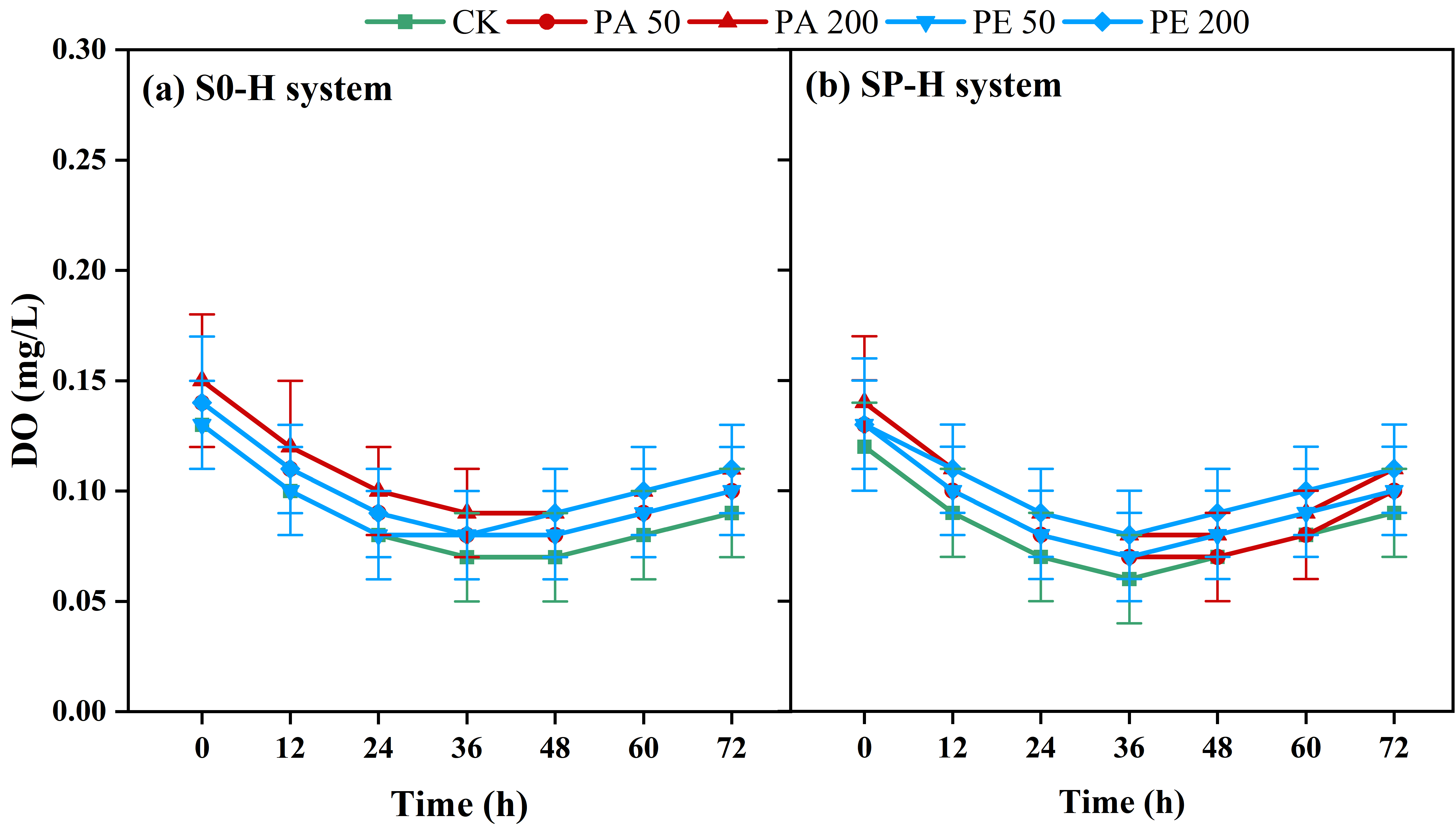


**Fig. S3** DO dynamics in the (a) S0-H and (b) SP-H incubation systems during 72 h of cultivation. DO remained below 0.20 mg/L in all treatments of both systems, with no significant treatment- or strain-dependent differences (*p* > 0.05), indicating that the divergent denitrification responses were not attributable to differences in oxygen availability. The error bars represent standard errors of the mean (SD) (n=3).


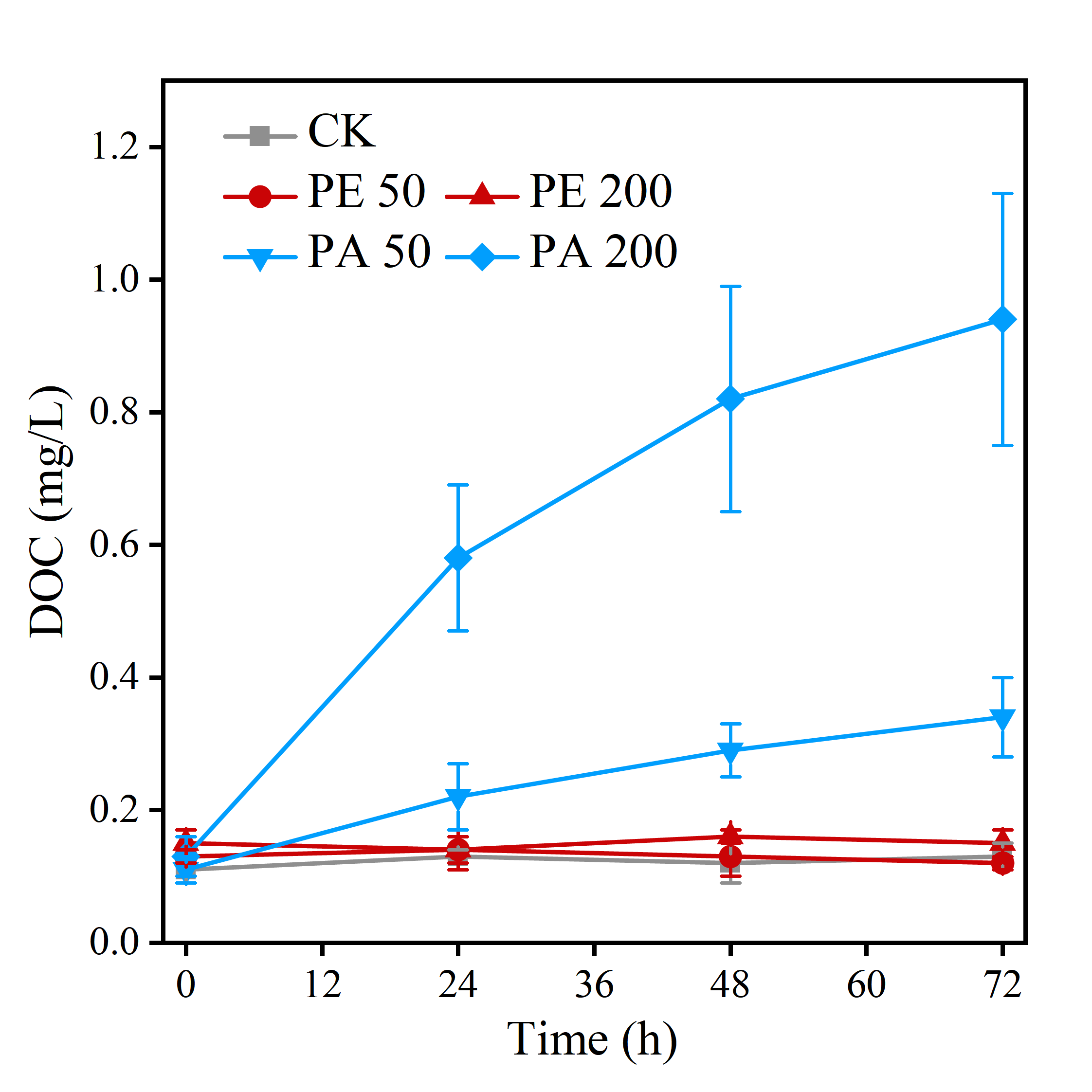


**Fig. S4** Dissolved organic carbon (DOC) released from sterile PA and PE MPs in the autotrophic medium over 72 h of incubation in the absence of bacterial inoculation. The error bars represent standard errors of the mean (SD) (n=3).


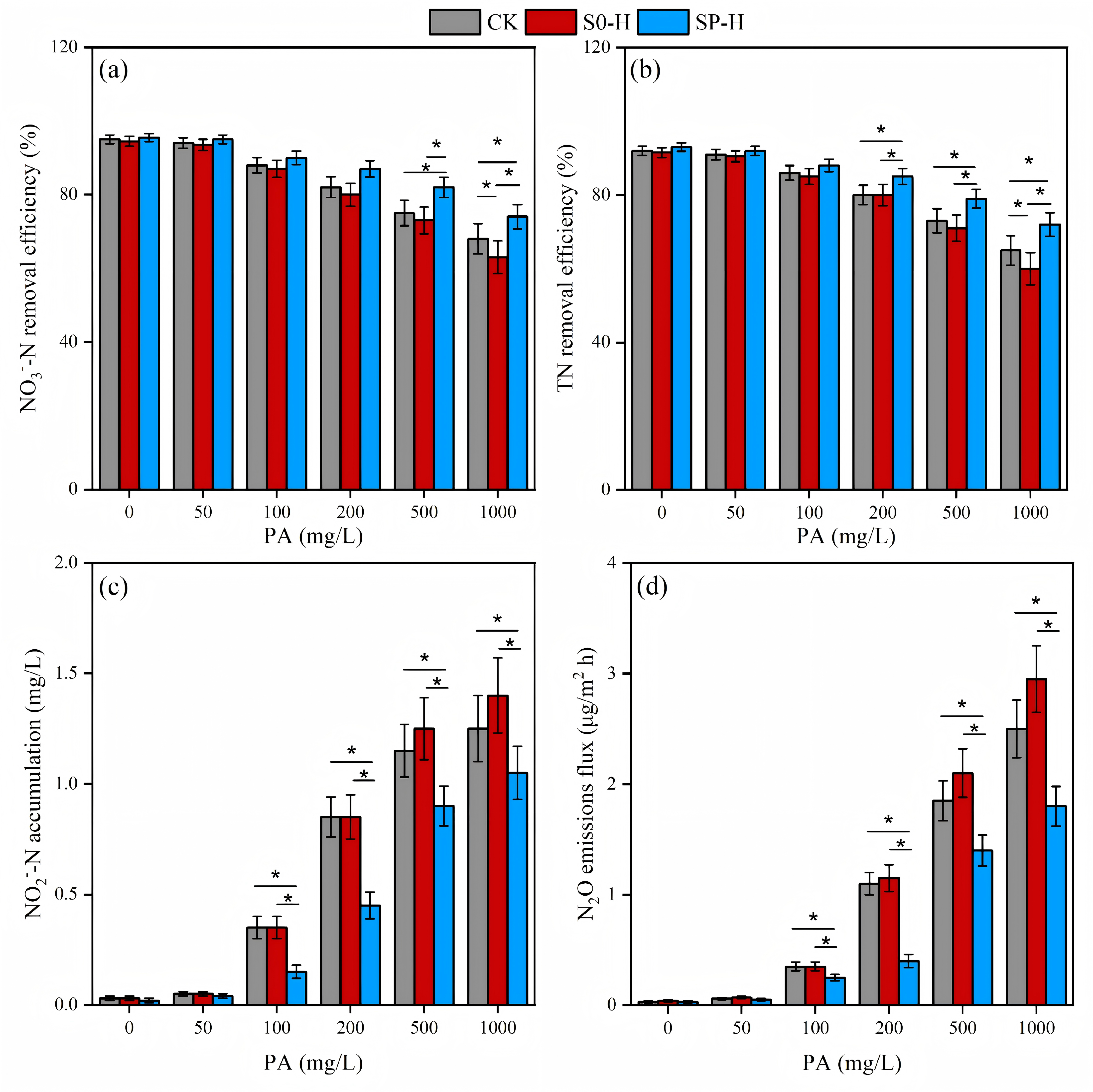


**Fig. S5** Preliminary mesocosm-scale verification of S0-H and SP-H bioaugmentation in a sulfur-based autotrophic constructed wetland simulation system (Fig. S1) under varying PA MP loadings. NO_3_^-^-N (a)/TN (b) removal efficiency, NO_2_^-^-N accumulation (c) and N_2_O emission flux (d). Treatments: CK (non-inoculated control), S0-H (inoculated with S0-H), and SP-H (inoculated with SP-H). Data are presented as mean ± SD (n = 3) and represent the averaged over 14–28 d after a 7 d inoculation and a 7 d transition. Asterisks indicate significant differences between groups (*, *p* < 0.05).

**Table S1** Carbon source utilization of strains S0-H and SP-H.

| **Carbon source** | **Concentration** | **S0-H** | **SP-H** |
| --- | --- | --- | --- |
| Glucose | 1.0 g/L | − | + |
| Sucrose | 1.0 g/L | − | + |
| Sodium acetate | 1.0 g/L | + | + |
| Sodium citrate | 1.0 g/L | + | + |
| Methanol | 1.0 g/L | − | + |
| Ethanol | 1.0 g/L | − | + |

**Note:** “+” indicates growth (OD_600_ > 0.1 after 7 d of incubation in the modified ATCC 1255 medium with the indicated carbon source as the sole substrate), hence utilization of the carbon source; “−” indicates no growth (OD_600_ ≤ 0.1). Each group was conducted in triplicate.

**Table S2** Quantitative analysis of cell viability based on CLSM Live/Dead staining images (Fig. 5) of strains S0-H and SP-H under MP stress.

| **Treatment** | **Viable**  **S0-H**  **cells (%)** | **Dead/damaged**  **S0-H**  **cells (%)** | **Viable**  **SP-H**  **cells (%)** | **Dead/damaged**  **SP-H**  **cells (%)** |
| --- | --- | --- | --- | --- |
| **CK** | 92.4 ± 2.1 | 7.6 ± 2.1 | 93.8 ± 1.8 | 6.2 ± 1.8 |
| **PA 50** | 64.2 ± 3.8 ** | 35.8 ± 3.8 ** | 87.5 ± 2.6 * | 12.5 ± 2.6 * |
| **PA 200** | 38.6 ± 4.5 *** | 61.4 ± 4.5 *** | 78.3 ± 3.1 ** | 21.7 ± 3.1 ** |
| **PE 50** | 87.3 ± 2.4 | 12.7 ± 2.4 | 91.6 ± 2.0 | 8.4 ± 2.0 |
| **PE 200** | 62.8 ± 3.6 ** | 37.2 ± 3.6 ** | 85.4 ± 2.8 * | 14.6 ± 2.8 * |

**Note:** Values represent means ± SD based on quantitative analysis of CLSM images using ImageJ (n = 5 random fields per treatment, each containing > 200 cells). Viable cells were identified by green fluorescence (SYTO 9 staining); dead or membrane-compromised cells were identified by red fluorescence (propidium iodide staining). Asterisks indicate significant differences between each MP treatment and the corresponding control (CK) (*, *p* < 0.05; **, *p* < 0.01; ***, *p* < 0.001).
